# Process Optimization of Polishing Titanium Alloy Material with a Pulsating Air Jet

**DOI:** 10.3390/ma16206813

**Published:** 2023-10-23

**Authors:** Lei Zhang, Chen Ding, Jianfa Bu, Zhirui Zhang, Yongguang Wang, Cheng Fan

**Affiliations:** 1 Jiangsu Provincial Key Laboratory of Advanced Robotics, College of Mechanical and Electrical Engineering, Soochow University, Suzhou 215021, China20215229117@stu.suda.edu.cn (J.B.);; 2 School of Mechanical and Electrical Engineering, Changchun University of Technology, Changchun 130012, China

**Keywords:** titanium alloy, pulsating air jet polishing, optimization, regression analysis

## Abstract

Titanium alloy is a widely used metal material, which can be applied in fields such as healthcare, petroleum exploration, aerospace, etc. In this paper, a new method for polishing the titanium alloy by a pulsating air jet is proposed. Compared with traditional abrasive jet polishing, this method has the advantages of simple structure, low nozzle wear, and high polishing flexibility. The working principle and material removal mechanism of the pulsating air jet polishing (PAJP) are introduced. Combined with orthogonal experiments, range analysis and variance analysis were used to find out the influence degree of each process parameter on the PAJP of titanium alloy, and the optimal level of each parameter was found. Through the experiments, a prediction model of surface roughness was established by regression analysis, and the predicted value was compared with the measured value. The maximum relative error of the prediction model was 10.3%, and the minimum relative error was 1.1%. The average relative error was 6.2%.

## 1. Introduction

As a high-strength material, titanium alloy has been widely used in the aerospace industry and the biomaterial manufacturing industry due to its excellent mechanical properties and good corrosion resistance [1,2,3]. However, titanium alloy is difficult to process, and the surface quality of titanium alloy after milling is poor. The polishing process is often used to remove milling marks and improve the surface quality of parts. In addition, polishing parameters have a direct impact on the residual stress and plastic deformation of the material surface; thus, the polishing process has attracted more and more attention from scholars [4,5,6]. In traditional contact polishing, Axinte et al. [7] systematically studied the abrasive belt polishing of Ti-6Al-4V. The results show that no obvious heat-affected zone is found on the workpiece surface. Guilherme et al. [8] studied the surface roughness of Ti-6Al-4V polished by different polishing methods. They concluded that the electrolytic polishing method can achieve better surface roughness for the titanium alloy than traditional polishing methods. Deng et al. [9] studied the residual stress on the surface of the titanium alloy workpiece after grinding and polishing, established a mathematical model of the surface residual stress, and verified the accuracy of the model through experiments. Liu et al. [10] optimized the abrasive belt polishing process of titanium alloy through the polishing experiments to obtain a higher surface quality. Yin et al. [11] studied the influences of polishing speed, feed rate, polishing depth, and particle size of the abrasive belt on the surface integrity after the belt grinding and polishing of titanium alloy.

The above-mentioned polishing methods are mainly traditional contact polishing. Due to the geometrical structure of the polishing tool, these methods are usually limited by the shape of the workpiece surface, and it is easy to leave tool marks on the workpiece surface [12,13]. Therefore, some scholars have carried out research on non-contact polishing in light of the above shortcomings. In the non-contact polishing method, the traditional tool head is replaced by a mixed fluid. The mixed fluid is composed of some flexible fluids and abrasives. It is driven by a power device at a high speed and high pressure to impact the workpiece surface. Abdel [14] established a mathematical model for the abrasive water jet polishing of ceramic materials, which predicted the maximum removal depth of polishing and was verified through experiments. Wang [15] established a mathematical model for the abrasive water jet polishing of alumina ceramics through dimensional analysis. Multiple linear regression analysis was performed on the experimental results to obtain a predictive model of its removal depth. The predicted results were in good agreement with the experimental results (the error was within 1%). Naresh et al. [16] constructed a quadratic polynomial model of the surface roughness for the abrasive water jet polishing of brass. They finally found out the optimal process parameters corresponding to the best surface quality through experiments. Fang et al. [17] studied the influence of the incidence angle on surface roughness, and a linear dependence of material removal on the working pressure was obtained. Mohamad et al. [18] established a surface roughness prediction model for the abrasive water jet polishing of Al7075-T6, and analyzed the surface roughness models corresponding to different initial conditions. Natarajan [19] extended the abrasive water jet polishing technique to the polishing of 304 stainless steel and deeply analyzed the three-dimensional surface topography of various polished surfaces. Liu et al. [20] studied the effect of process parameters on the removal depth and surface roughness in the abrasive water jet polishing of alumina ceramics. The influence of each process parameter was studied through the variance analysis technique. Li et al. [21] built a numerically controlled experimental system of abrasive jet polishing and a flat optical glass with a diameter of 20 mm was polished. After two iterations of polishing, the roughness of the surface was within 2 nm. Li et al. [22] processed the micropores on the glass surface with a micro-abrasive air jet. They found that the profiles of the micropores would change with the flow rate of abrasive particles and the air pressure. Lari and Papini [23,24] established a mathematical model for the surface evolution during the processing of brittle materials by a micro-abrasive air jet, which could accurately predict the profile topography of the cross-section. However, the problems of nozzle clogging, nozzle wear, and abrasive agglomeration in abrasive water jet polishing have not been completely solved, which restricts the application of the water jet polishing process in the polishing of some large curved surfaces.

The difference between the pulsating air jet polishing (PAJP) proposed In this paper and the traditional abrasive jet polishing is that the abrasive particles are no longer sprayed from the nozzle, but are mixed in the abrasive fluid in the container. After the air is pressurized by the compressor, the abrasive particles in the abrasive fluid are driven by a high-speed air jet beam from the nozzle. Then the abrasive particles are accelerated to impact the workpiece surface at a high speed to complete material removal. In addition, countless tiny bubbles will be generated when the gas is jetted. These bubbles will collapse and shatter after colliding with the workpiece, and instantly generate a cavitation effect. The cavitation effect creates strong shock waves and micro-jets that can accelerate material removal.

In this paper, the main process parameters include jet pressure, jet angle, jet distance, abrasive concentration, and abrasive particle size. The effect of the above factors on polishing quality was analyzed by orthogonal experiments, and the optimal process parameters when polishing titanium alloy by PAJP were found. The surface roughness model of titanium alloy was obtained by multiple linear regression analysis, and error analysis was carried out.

## 2. PAJP Experimental Platform

**Figure 1:**
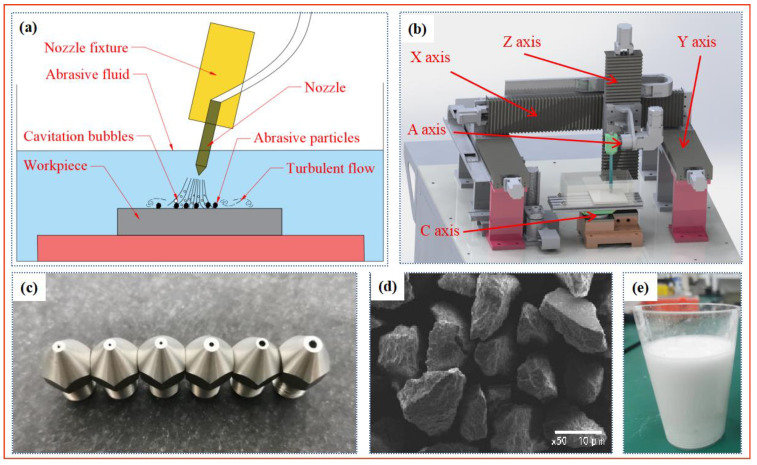
Composition of PAJP polishing system [25]. (**a**) Polishing principle of PAJP, (**b**) device of PAJP, (**c**) conical–straight nozzle, (**d**) microscopic topography of alumina abrasives, and (**e**) alumina abrasive fluid.

Figure 1a,b show the working principle of PAJP and the corresponding polishing machine tool. As shown in Figure 1a, the nozzle and the workpiece are completely submerged in the abrasive fluid. The abrasive fluid is driven by the pulsating air to polish the surface of the material [25]. In order to facilitate the observation of the flow field under working conditions, the container tank is made of transparent acrylic plates. The size of the container is 300 × 150 × 100 mm^3^. The diameter of the jet tube is 8 mm. The air jet tool system is fixed on the A-axis of the machine tool, and the polishing path of the nozzle is controlled by the XYZ three-axis linkage of the machine tool. The jet angle is adjusted by controlling the A-axis of the machine tool, and the rotation of the C-axis turntable can realize the rotation of the workpiece. As shown in Figure 1c, because the impact force of the conical–straight nozzle is stronger and more stable than that of other nozzles, the conical–straight nozzle is selected in this paper. The diameter of the nozzle determines the impact area and the polishing efficiency. 

The abrasive fluid is composed of the abrasives, base fluid, suspending agent, and dispersing agent in a certain proportion. The main function of the base fluid is to mix the abrasive with other fluids. The function of the suspending agent is to prevent the abrasive from settling at the bottom and to prevent the powder from hardening into lumps [25]. The function of the dispersing agent is to make the abrasive evenly dispersed in the base fluid. The proportion of each component in the abrasive fluid determines the polishing quality and polishing efficiency. The effect of the solution was observed by adding different reagent contents. The proportion of the suspending agent is determined to be 1% of the water mass fraction, and the proportion of the dispersing agent is determined to be 0.8% of the water mass fraction. Figure 1e shows the abrasive fluid containing the abrasives, base fluid, suspending agent, and dispersing agent.

**Figure 2:**
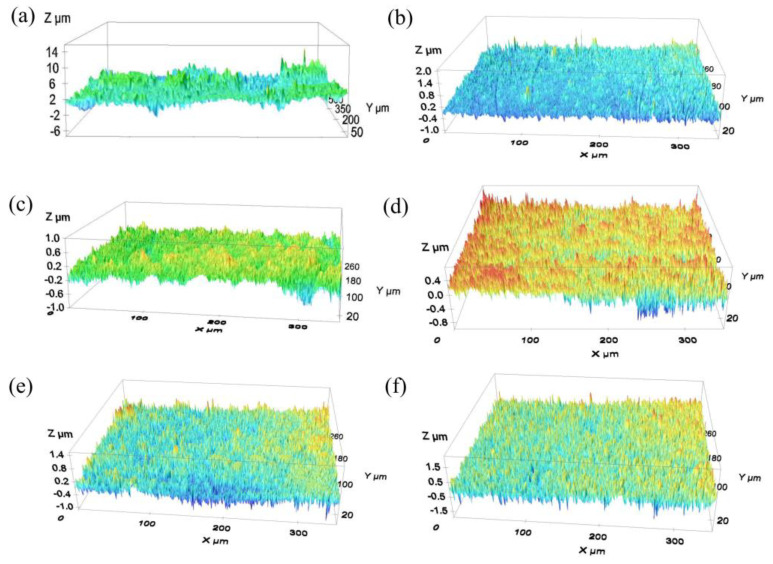
Surface topography of titanium alloy sample after submerged air jet polishing: (**a**) initial surface, (**b**) surface polished with 5 μm alumina abrasives, (**c**) 10 μm alumina abrasives, (**d**) 15 μm alumina abrasives, (**e**) 15 μm silicon carbide abrasives, and (**f**) 15 μm diamond abrasives.

**Figure 3:**
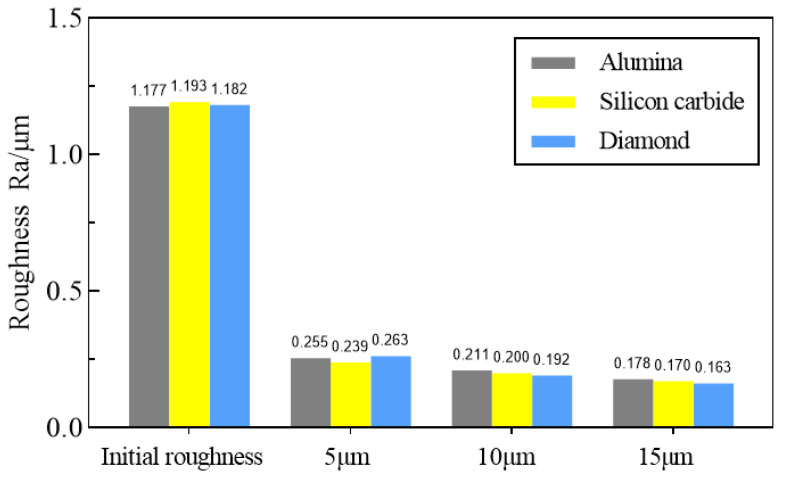
Surface roughness after polishing with different types and particle sizes of abrasive [25].

The choice of the type of abrasive is related to the hardness of the material. Generally, when polishing a rough surface, the hardness of the abrasive is preferred to be greater than that of the workpiece. In this paper, three different abrasives, alumina (Al_2_O_3_), silicon carbide (SiC), and diamond were initially selected for the pulsating air jet polishing experiments. Their Mohs hardness are higher than titanium alloy. The average particle size of each abrasive is 5 μm, 10 μm, and 15 μm, respectively. The concentration of abrasive fluid is 6%. The other parameters include an air pressure of 0.4 MPa, injection distance of 4 mm, injection angle of 30°, and processing time of 30 min. Figure 2 shows the surface topographies of the titanium alloy samples before and after pulsating air jet polishing. As shown in Figure 3, within 30 min of polishing, the surface roughness after polishing with 5 μm, 10 μm, and 15 μm aluminum oxide polishing fluid is reduced to 0.255 μm, 0.211 μm, and 0.178 μm, respectively. The surface roughness after polishing with 5 μm, 10 μm and 15 μm diamond polishing fluid is reduced to 0.239 μm, 0.200 μm, and 0.170 μm respectively. The surface roughness after polishing with 5 μm, 10 μm, and 15 μm silicon carbide polishing fluid is reduced to 0.263 μm, 0.192 μm, and 0.163 μm respectively. The larger the abrasive particle size is, the faster the material removal is. With the increase in abrasive particle size, the polishing effect of three abrasives on the titanium alloy is obvious. This shows that the abrasive particle size has a great influence on the polishing quality. Through the experiments, it is found that the polishing effect of the three selected abrasives is not significantly different; thus, this paper selects the alumina abrasive with the highest cost performance to carry out various polishing experiments and process optimization.

Since the experiments in this paper are aimed at fine polishing after the rough machining of the material surface, the range of abrasive particle size selected in this paper is 3 μm–15 μm. Figure 1d shows the microscopic topography of the alumina abrasives under the scanning electron microscope. It can be seen from the figure that most of the abrasive particles have irregular polygonal shapes.

**Figure 4:**
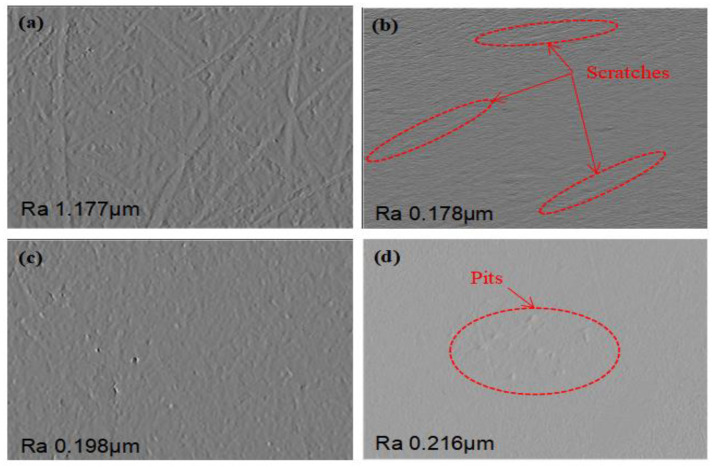
Surface morphology after polishing with different jet angles. (**a**) Before polishing, (**b**) polished with jet angle of 30°, (**c**) polished with jet angle of 60°, and (**d**) polished with jet angle of 90°.

As shown in Figure 4a, a large number of milling marks are left on the workpiece before polishing. After polishing with different jet angles, the surface roughness is improved greatly, as shown in Figure 4b–d. In the process of polishing, there are two main methods for material removal according to the jet angle: one is micro-cutting removal and the other is extrusion removal. When the jet angle is small, most of the material is removed by micro-cutting removal [25]. As shown in Figure 4b, there are many scratches caused by micro-cutting removal. When the jet angle is larger, most of the material is removed by extrusion removal. As shown in Figure 4d, there are many pits caused by extrusion removal [26,27].

Through some single-factor experiments, the range of each process parameter is preliminarily determined as follows: the jet pressure is 0–1 MPa, the jet distance is 4–10 mm, the jet angle is 30–90°, the abrasive concentration is 4–10%, and the particle size is 4–15 μm. Next, the optimal combination of process parameters for polishing titanium alloys will be determined within this range.

## 3. Design of Orthogonal Experiment

To obtain a higher surface quality for titanium alloys under PAJP, the surface roughness is taken as the index to measure the quality of the polishing. The influence of process parameters on the roughness is analyzed through orthogonal experiments, and the optimal parameter combination is found. In this paper, a standard orthogonal table L_16_(4^5^) with five factors and four levels is adopted. The factors selected in the orthogonal experiments and their levels are shown in Table 1. 

**Figure 5:**
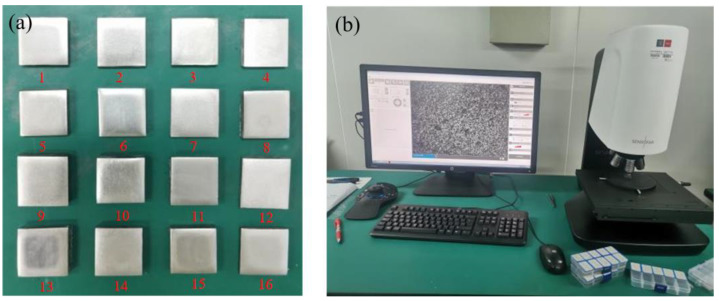
Experimental samples and measuring instrument. (**a**) Titanium alloy samples and (**b**) Spain Sensofar white light interferometer (PLu Neox, Barcelona, Spain).

Table 2 shows the orthogonal experimental design including 16 experiments. Each experiment in Table 2 represents a combination of the process parameters. For example, Experiment No.2 (A_1_B_2_C_2_D_2_E_2_) in Table 2 represents the following parameter settings: the jet pressure is set as 0.2 MPa, the jet distance is set as 6 mm, the jet angle is set as 45°, the abrasive concentration is set as 6%, and the abrasive particle size is set as 6 μm. The samples required in 16 groups of experiments are shown in Figure 5a. The size of each sample is 20 mm × 20 mm × 5 mm and the material of the samples is titanium alloy. The initial average surface roughness is 1.563 μm and the polishing time is 30 min with an on–off frequency of once per second. As shown in Figure 5b, the Spain Sensofar white light interferometer is used to measure the surface roughness of the sample. 

## 4. Experimental Results and Discussion

### 4.1. Range Analysis

According to the orthogonal table, 16 groups of experiments were conducted. The surface roughness measured after the polishing is in the last column of Table 2.

In Table 2, *K*_i_ (*i* = 1, 2, 3, 4) represents the sum of roughness with experiment number *i* in any column, and *k*_i_ (*i* = 1, 2, 3, 4) corresponds to the arithmetic average of *K*_i_. The optimal level of factors can be judged by the value of *k*_i_. In this experiment, the level corresponding to the minimum value of *k*_i_ is the optimal level. *R* represents the degree of dispersion of data and its calculation formula is

(1) $$R=\max\left\{k_{1},k_{2},k_{3}\right\}-\min\left\{k_{1},k_{2},k_{3}\right\}$$

**Figure 6:**
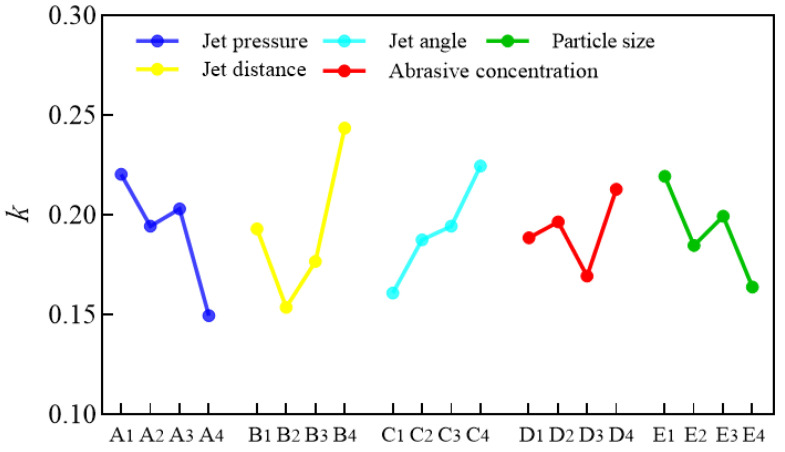
Line chart of *k* values corresponding to the different factors.

The value of *R* reflects the influence of different factors on roughness. The larger the *R* is, the greater the influence of this factor on roughness. Therefore, according to the results in Table 3, the influence degree of the factors from large to small is B, A, C, E, and D (jet distance, jet pressure, jet angle, particle size, and abrasive concentration). In order to intuitively reflect the influences of jet pressure, jet distance, jet angle, particle size, and abrasive concentration on the surface roughness, the line chart of the factors is drawn in Figure 6. The lowest point of each curve in the figure can be identified as the optimal parameter combination of this experiment. Therefore, the optimized horizontal combination is A_4_B_2_C_1_D_3_E_4_, that is, the jet pressure is 0.8 MPa, the jet distance is 6 mm, the jet angle is 30°, the abrasive concentration is 8%, and the abrasive particle size is 15 μm.

**Figure 7:**
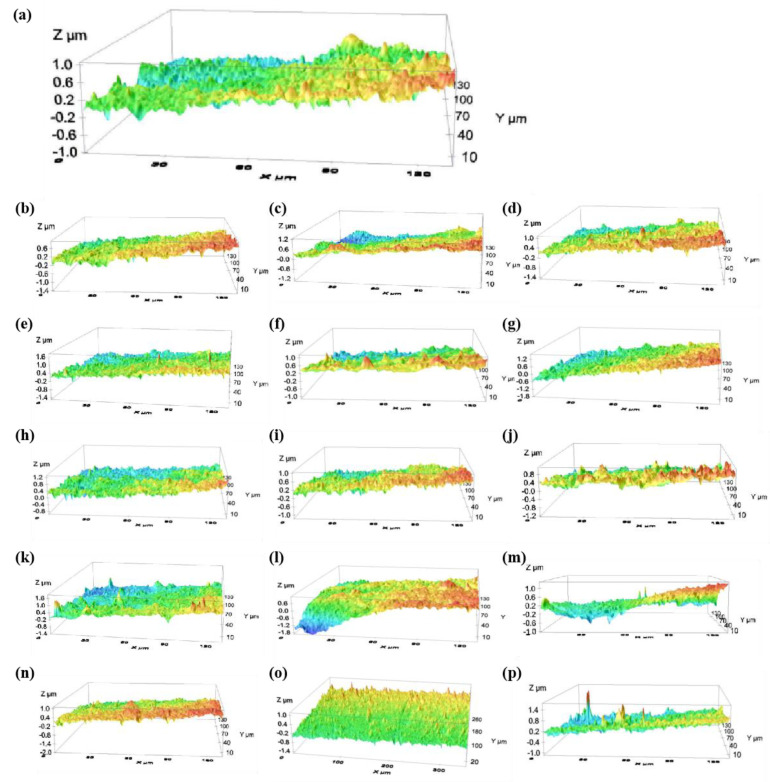
Three-dimensional topography of each group in the orthogonal experiments. (**a**) sample 1, (**b**) sample 2, (**c**) sample 3, (**d**) sample 4, (**e**) sample 5, (**f**) sample 6, (**g**) sample 7, (**h**) sample 8, (**i**) sample 9, (**j**) sample 10, (**k**) sample 11, (**l**) sample 12, (**m**) sample 13, (**n**) sample 14, (**o**) sample 15, (**p**) sample 16.

The three-dimensional topographies of the polished surfaces in the orthogonal experiments are shown in Figure 7, and Figure 7a–p correspond to the results of experiments 1–16, respectively. The average roughness of the 16 samples is 0.192 μm, which is significantly lower than that before polishing. The lowest surface roughness of the titanium alloy obtained by experiment No.14 is *Ra* = 0.104 μm.

### 4.2. Variance Analysis

In order to judge the significance of the influence of various factors on the experimental results, SPSS was used for the corresponding convenient analysis of the experimental results in the above table, as shown in Table 3. It can be concluded that A (jet pressure), B (jet distance), and C (jet angle) have a significant effect on the polished surface roughness of the titanium alloy samples, while D (abrasive concentration) and E (particle size) have an insignificant effect on the polished surface roughness of the titanium alloy samples. In addition, it can be seen from the value of the partial Eta square that the larger the value is, the larger the proportion of influence is; thus, the degree of influence from large to small is B > A > C > E > D. This is consistent with the range analysis.

## 5. Surface Roughness Prediction

### 5.1. Regression Model

During the PAJP process, there are many process parameters that affect the surface quality of the workpiece. In order to reduce polishing costs and improve polishing efficiency, it is very important to establish a surface roughness prediction model through regression analysis. It can be assumed that the surface roughness of Ti-6Al-4V after polishing has the following relationship with five parameters, namely jet pressure (*P*), jet distance (*S*), jet angle (*α*), abrasive concentration (*W*), and particle size (*D*), which gives

(2) $$Ra=K\cdot P^{\beta_{1}}\cdot S^{\beta_{2}}\cdot \alpha^{\beta_{3}}\cdot W^{\beta_{4}}\cdot D^{\beta_{5}}$$

where *K* is the coefficient related to the polishing system and *β*_i_ is the coefficient to be determined.

Taking the logarithm of both sides of Equation (2), it can be further modified as

(3) $$\ln Ra=\ln K+\beta_{1}\ln P+\beta_{2}\ln S+\beta_{3}\ln\alpha +\beta_{4}\ln W+\beta_{5}\ln D$$

Let *Y* = ln*Ra*, *β*_0_ = ln*K*, *x*_1_ = ln*P*, *x*_2_ = ln*S*, *x*_3_ = ln*α*, *x*_4_ = ln*W*, *x*_5_ = ln*D*, Equation (3) is transformed into

(4) $$Y=\beta_{0}+\beta_{1}x_{1}+\beta_{2}x_{2}+\beta_{3}x_{3}+\beta_{4}x_{4}+\beta_{5}x_{5}$$

Equation (4) is a multiple linear equation of *Y* with respect to *x*_1_, *x*_2_, *x*_3_, *x*_4_, and *x*_5_, which can be solved by multiple linear regression analysis. The data in Table 3 is imported into SPSS for multiple linear regression analysis, and the results are shown in Table 4.

**Figure 8:**
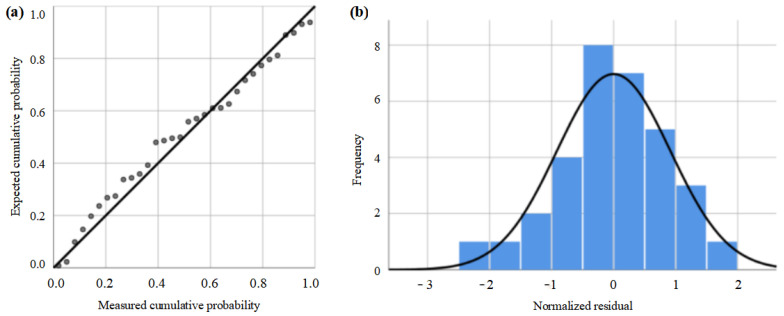
Normal independence test results for regression model. (**a**) Normalized residual distribution, (**b**) histogram of residuals.

In Table 4, R square can be used to determine the degree of fit of the regression equation, with values ranging from 0 to 1. The closer the value of R square is to 1, the better the degree of fit [28]. If the R square equals 0.60, it can be readily assumed that 60% of the variables in Y are explained by X in the fitting formula. As can be seen from Table 4, the significant value of the regression model is less than 0.0001 and the R square value of the model is 0.747, indicating that the prediction model is relevant [28]. By fitting the roughness regression model and analyzing the variance of the parameter, the residual distribution is shown in Figure 8. As shown in Figure 8a, the scattered points are distributed on both sides of the central line and the deviation from the experimental data is small, indicating that the regression model has a high degree of fitting. As shown in Figure 8b, the residual surface roughness after polishing by PAJP satisfies the normal distribution, indicating that the regression prediction model has high accuracy and can be used for further modeling optimization. Since *β*_0_ = ln*K*, *K* = 0.040. *β*_1_–*β*_5_ can be obtained from the coefficient column, their values are −0.217, 0.171, 0.279, 0.073, and −0.109, respectively. The final regression equation is

(5) $$Ra=0.040\cdot P^{-0.217}S^{0.171}\alpha^{0.279}W^{0.073}D^{-0.109}$$

### 5.2. Error Analysis

The prediction model based on statistical regression analysis is only a theoretical mathematical model, and the prediction effect of the model needs to be verified by actual data. From the previous range analysis and variance analysis, it can be seen that the main factors affecting the surface roughness of titanium alloy are jet pressure, jet distance, and jet angle, and the influence of particle size and abrasive concentration is relatively small. Therefore, in order to facilitate the experiment, the abrasives are all alumina micro-powders with an average particle size of 10 μm and other parameters are random. A comparison of the experimental roughness values with the predicted values of the regression model is shown in Table 5.

Then, the error is calculated and analyzed to judge the quality of the prediction model according to the size of the error. For the error between the measured value and the predicted value, the calculation method of absolute error and relative error is introduced. 

Absolute error is the absolute value of the difference between the predicted value (*Xp*) and the measured value (*Xr*), which is expressed by *e*_1_ as

(6) $$e_{1}=\left|X_{p}-X_{r}\right|$$

Relative error is the percentage of absolute error in the measured value, which is expressed by *e*_2_ as

(7) $$e_{2}=\left|\frac{X_{p}-X_{r}}{X_{r}}\right|\times 100\%$$

Mean relative error is the mean of relative error, which is expressed by $\bar{e_{2}}$ as

(8) $$\bar{e_{2}}=\frac{\sum_{i=1}^{10}e_{2}}{10}$$

The error analysis results are shown in Table 6.

As can be seen from Table 6, the maximum relative error is 10.3% and the minimum relative error is 1.1%. The average relative error is 6.2%, which is within the acceptable range [28]. Therefore, the prediction model of roughness for polishing titanium alloy is reliable. In the study of submerged air jet polishing, how to improve the precision and efficiency of polishing is the main research direction. The polishing of titanium alloy materials carried out in this paper is only a small part of all the research involving pulsating air jet polishing, and there are many more complicated problems that need to be further studied. 

## 6. Conclusions

A new method of PAJP for polishing titanium alloy (Ti-6Al-4V) was proposed. Through the orthogonal experiments, the main process parameters of PAJP affecting the surface roughness of titanium alloys were explored. The results show that the influence of various process parameters on the surface roughness of titanium alloys is in the order of jet distance > jet pressure > jet angle > particle size > abrasive concentration. The process of PAJP for polishing titanium alloy was optimized by the range analysis. The optimal process parameters are: jet pressure 0.8 MPa, jet distance 6 mm, jet angle 30°, abrasive concentration 8%, and particle size 10 μm.

The prediction model of surface roughness was obtained by multiple linear regression analysis. According to the results, the maximum relative error is 10.3%, the minimum relative error is 1.1%, and the average relative error is 6.2%, which indicates that the prediction model of roughness for polishing titanium alloy is reliable. With the optimal process parameters and prediction model of surface roughness for the PAJP, the next step will be to study how to use this method to polish the entire surface and explore how to effectively improve the roughness and form accuracy of the entire surface. 

## Figures and Tables

**Table 1:** Factors and levels graph.

	Factors	A	B	C	D	E
Levels		Jet Pressure/MPa	Jet Distance/mm	Jet Angle/°	Abrasive Concentration/%	Particle Size/μm
1		0.2	4	30	4	4
2		0.4	6	45	6	6
3		0.6	8	60	8	10
4		0.8	10	90	10	15

**Table 2:** L_16_(4^5^) orthogonal experimental design and results.

Experiment Number	A	B	C	D	E	Roughness (μm)
	Jet Pressure/MPa	Jet Distance/mm	Jet Angle/°	Abrasive Concentration/%	Particle Size/μm	
1	0.2	4	30	4	4	0.215
2	0.2	6	45	6	6	0.155
3	0.2	8	60	8	10	0.193
4	0.2	10	90	10	15	0.319
5	0.4	4	45	8	15	0.162
6	0.4	6	30	10	10	0.154
7	0.4	8	90	4	6	0.181
8	0.4	10	60	6	4	0.281
9	0.6	4	60	10	6	0.200
10	0.6	6	90	8	4	0.203
11	0.6	8	30	6	15	0.155
12	0.6	10	45	4	10	0.255
13	0.8	4	90	6	10	0.196
14	0.8	6	60	4	15	0.104
15	0.8	8	45	10	4	0.179
16	0.8	10	30	8	6	0.120
*K*_1_	0.882	0.773	0.644	0.755	0.878	
*K*_2_	0.778	0.616	0.751	0.787	0.656	
*K*_3_	0.813	0.708	0.778	0.678	0.798	
*K*_4_	0.599	0.975	0.899	0.852	0.740	
*k*_1_	0.2205	0.19325	0.161	0.18875	0.2195	
*k*_2_	0.1945	0.154	0.18775	0.19675	0.185	
*k*_3_	0.20325	0.177	0.1945	0.1695	0.1995	
*k*_4_	0.14975	0.24375	0.22475	0.213	0.164	
*R*	0.07075	0.08975	0.06375	0.0435	0.0555	
Optimal levels	A_4_	B_2_	C_1_	D_3_	E_4_	

**Table 3:** Inter-subjective effect test.

Items	Sum of Squares	Degrees of Freedom	Mean Square	F	Significant	Partial Eta Square
Correction model	0.76 ^a^	15	0.005	3.943	0.002	0.747
Intercept	0.695	1	0.695	539.077	0.000	0.964
Jet pressure (A)	0.019	3	0.006	4.913	0.010	0.324
Jet distance (B)	0.017	3	0.006	4.394	0.016	0.392
Jet angle (C)	0.025	3	0.008	6.450	0.003	0.297
Abrasive concentration (D)	0.008	3	0.003	2.166	0.124	0.096
Particle size (E)	0.009	3	0.003	2.210	0.118	0.104
Error	0.026	20	0.001			
Total	1.564	36				

^a^ represents a series of infinite acyclic numbers.

**Table 4:** Regression analysis results.

Model	Non Normalized Coefficient		Normalized Coefficient	t	Significant	Sum of Square
	Beta	Error	Beta			
Constant	−3.208	0.051		3.392	0.002	
Jet pressure	−0.217	0.041	−0.446	−3.325	0.002	
Jet distance	0.171	0.004	0.227	1.691	0.101	
Jet angle	0.279	0.000	0.470	3.500	0.001	
Abrasive concentration	0.073	0.004	−0.055	−0.412	0.683	
Particle size	−0.109	0.002	−0.100	−0.745	0.462	
Regression					0.000 ^a^	0.047
Residual						0.055
Total						0.102
R square						0.747

^a^ represents a series of infinite acyclic numbers.

**Table 5:** Comparison of measured roughness and predicted roughness.

No.	Jet Pressure (MPa)	Jet Distance (mm)	Jet Angle (°)	Abrasive Concentration (%)	Particle Size (μm)	*Xp* (μm)	*Xr* (μm)
1	0.5	5	50	5	10	0.159	0.143
2	0.2	2	35	2	10	0.141	0.148
3	0.3	3	40	3	10	0.178	0.182
4	1.0	10	90	10	10	0.204	0.216
5	0.1	1	20	1	10	0.236	0.204
6	0.6	6	55	6	10	0.165	0.179
7	0.8	8	70	8	10	0.178	0.163
8	0.7	7	60	7	10	0.169	0.183
9	0.9	9	80	9	10	0.185	0.187
10	0.4	4	45	4	10	0.154	0.142

**Table 6:** Error analysis of regression model.

No.	*Xp* (μm)	*Xr* (μm)	Absolute Error	Relative Error (%)	Average Relative Error (%)
1	0.159	0.143	0.008	5.3	6.2
2	0.141	0.148	0.007	4.7	
3	0.178	0.182	0.004	2.2	
4	0.204	0.216	0.012	5.6	
5	0.236	0.204	0.022	10.3	
6	0.165	0.179	0.014	7.8	
7	0.178	0.163	0.015	9.2	
8	0.169	0.183	0.014	7.7	
9	0.185	0.187	0.002	1.1	
10	0.154	0.142	0.012	8.5	

## Data Availability

The data that support the findings of this study are available from the corresponding author.
